# Supplementary material for: High and specific diversity of protists in the deep-sea basins dominated by diplonemids, kinetoplastids, ciliates and foraminiferans
Source: Commun Biol. 2021 Apr 23;4:501. doi: 10.1038/s42003-021-02012-5 (PMC8065057; doi:10.1038/s42003-021-02012-5)
Supplement: Supplementary file 2 — Supplementary Information [file 42003_2021_2012_MOESM2_ESM.pdf]

SUPPLEMENTARY INFORMATION FOR

High and specific diversity of protists in the deep-sea basins dominated by diplonemids, kinetoplastids, ciliates and foraminiferans

Alexandra Schoenle[1], Manon Hohlfeld[1], Karoline Hermanns[1], Frédéric Mahé[2,3], Colomban de Vargas[4,5], Frank Nitsche[1], Hartmut Arndt[1]**

[1] *University of Cologne, Biocenter, Institute for Zoology, General Ecology, Zülpicher Straße 47b, 50674 Cologne, Germany.*

[2] *CIRAD, UMR BGPI, F-34398, Montpellier, France.*

[3] *BGPI, Univ Montpellier, CIRAD, IRD, Montpellier SupAgro, Montpellier, France.*

[4] *CNRS, Sorbonne Université, Station Biologique de Roscoff, UMR7144, ECOMAP -Ecology of Marine Plankton, 29680 Roscoff, France*

[5] *Research Federation for the study of Global Ocean Systems Ecology and Evolution, FR2022/ Tara GOSEE, Paris, France*

*Corresponding authors

Supplementary Figures and Tables

Supplementary Figures 1 to 10

Supplementary Tables 1 and 2

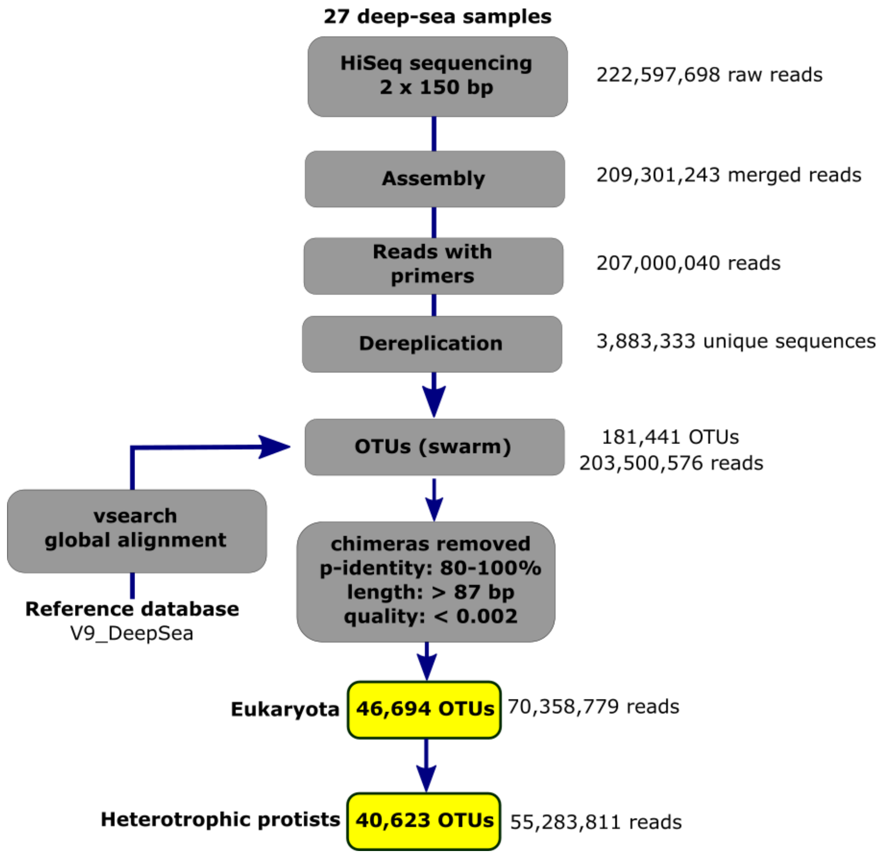

Supplementary Figure 1 | Bioinformatic pipeline. Raw V9 rDNA reads from 27 sediment samples taken at 20 stations from 11 deep-sea basins/regions were merged and only used for downstream analyses, when they contained both primers and no ambiguous N's. After de-replication, unique sequences (metabarcodes) with more than two reads were clustered into operational taxonomic units (OTUs) using the Swarm algorithm. A taxonomic path of the OTU representatives was assigned using Stampa [67]. After removal of chimeras, OTUs with less than 80% p-identity, smaller than 87 bp and OTUs with a minimum expected error >0.002 led to a final eukaryotic dataset of 46,694 OTUs of which 40,623 OTUs could be assigned to heterotrophic protists.

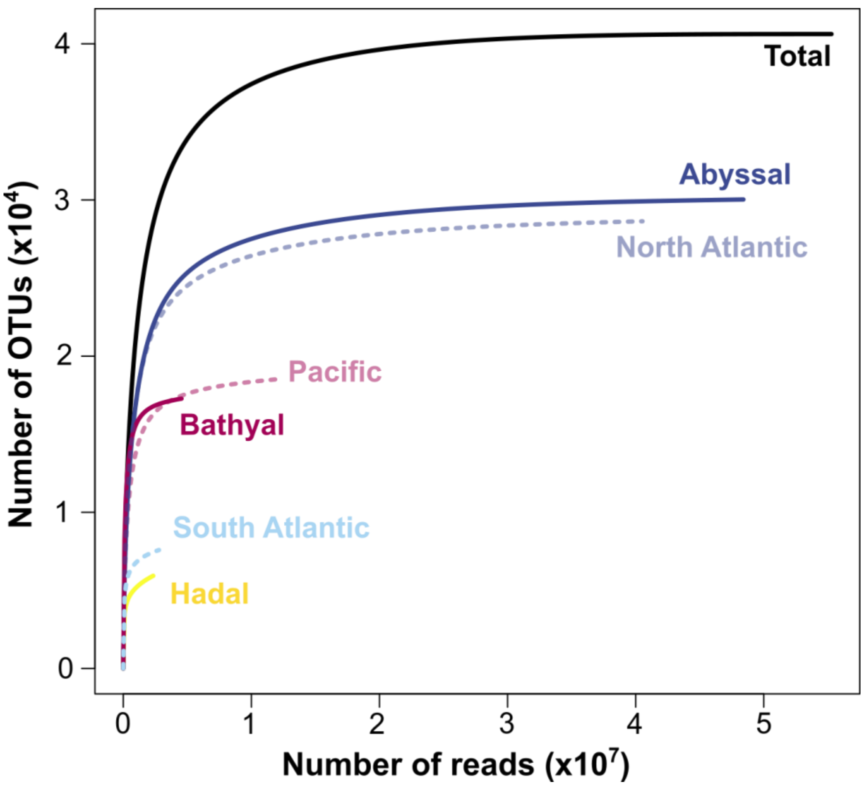

Supplementary Figure 2 | Assessment of deep-sea protist richness. V9 rDNA heterotrophic protist OTUs rarefaction curves from either region dependent (North Atlantic, South East Atlantic and Pacific Ocean) or depth dependent (bathyal, abyssal, hadal) clustering of all stations.

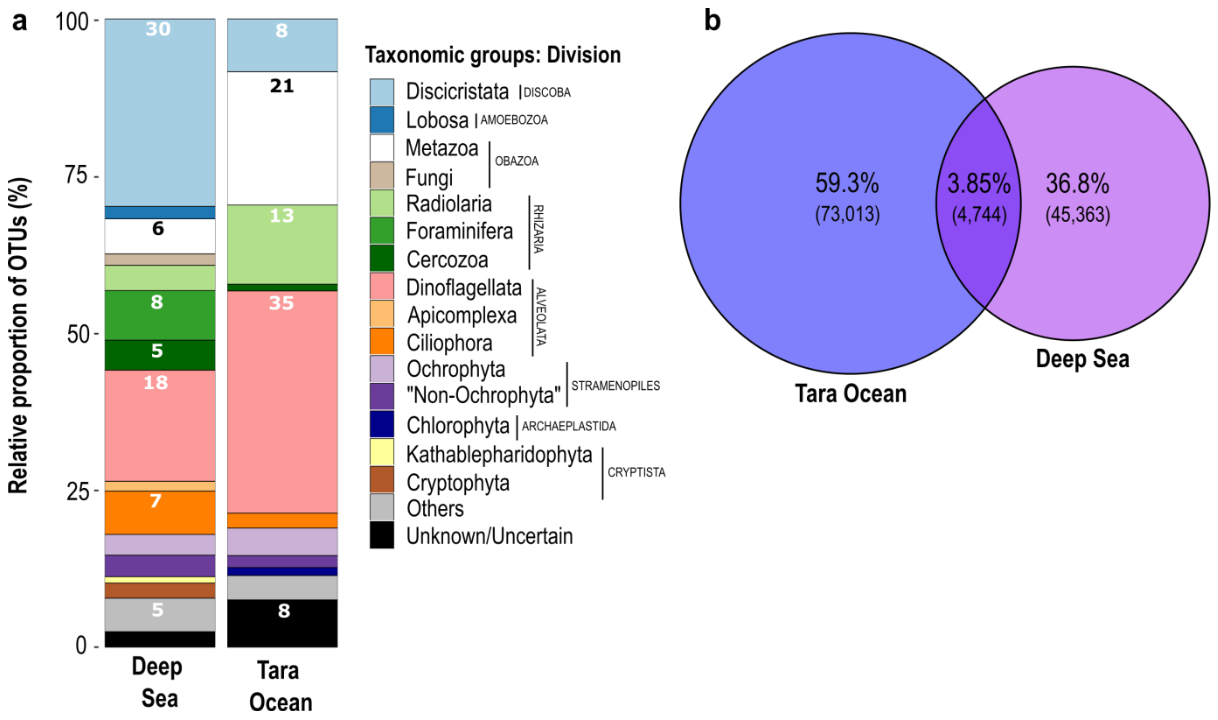

Supplementary Figure 3 | Comparison of eukaryotic species richness from surface waters and the deep ocean. (**a**) Relative proportion of eukaryotic OTUs (total of 123,120 OTUs) from the Tara Ocean data set and our deep-sea dataset blasted against our reference database V9_DeepSea [33]. Taxonomic division levels are only separately shown, when the number of OTUs reached more than 1% within one of the two environments. Otherwise, OTUs were clustered together into “Others”. Unknown/Uncertain” OTUs have been either assigned to several taxonomic division levels or to sequences taxonomically assigned only to Eukaryota. (**b**) Venn-diagram showing the number of unique and shared OTUs between sampled surface waters within the Tara Ocean project (47 stations, total 77,757 OTUs) and the sediment samples from the deep sea (20 stations, total of 50,107 OTUs).

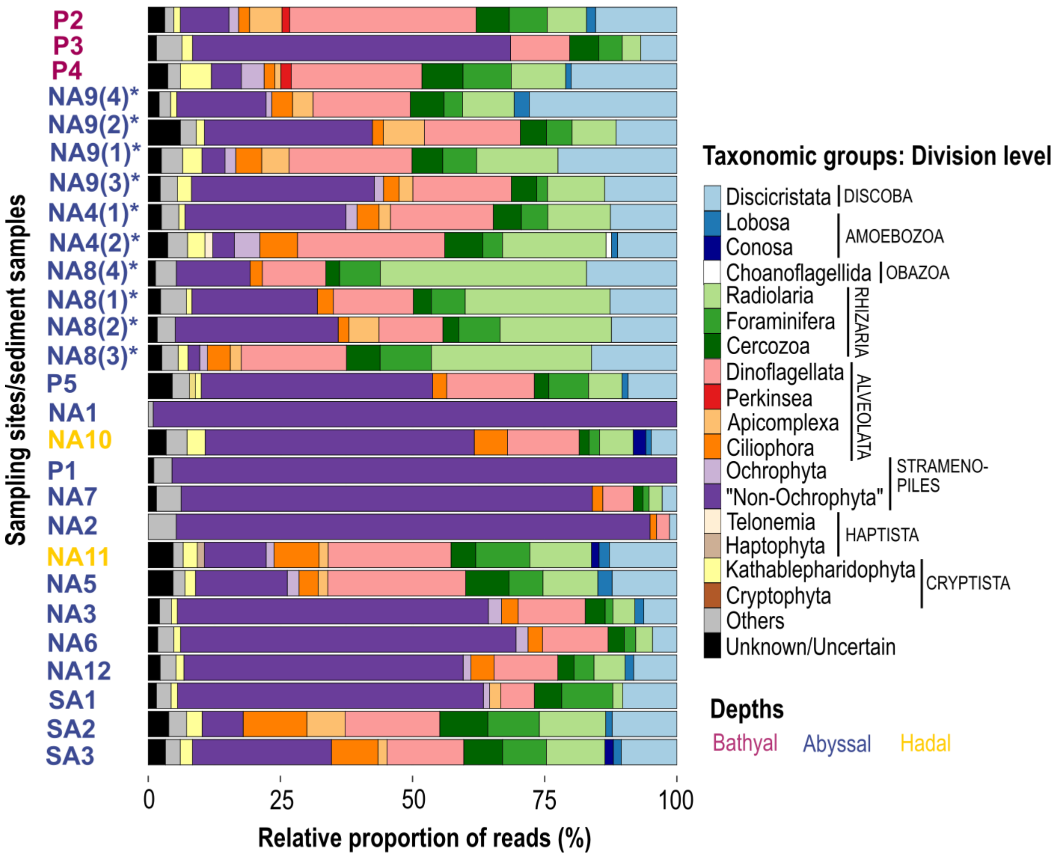

Supplementary Figure 4 | Community composition of deep-sea heterotrophic protists. Relative proportion of reads within the 27 deep-sea sediment samples related to the major taxonomic protist groups. Taxonomic groups (corresponding to division level in the PR2 database classification) are only separately shown, when the number of reads reached more than 1% within each sample. Otherwise, reads were clustered together into "Others". Unknown/Uncertain" reads have been either assigned to several taxonomic division levels or to sequences taxonomically assigned only to Eukaryota.

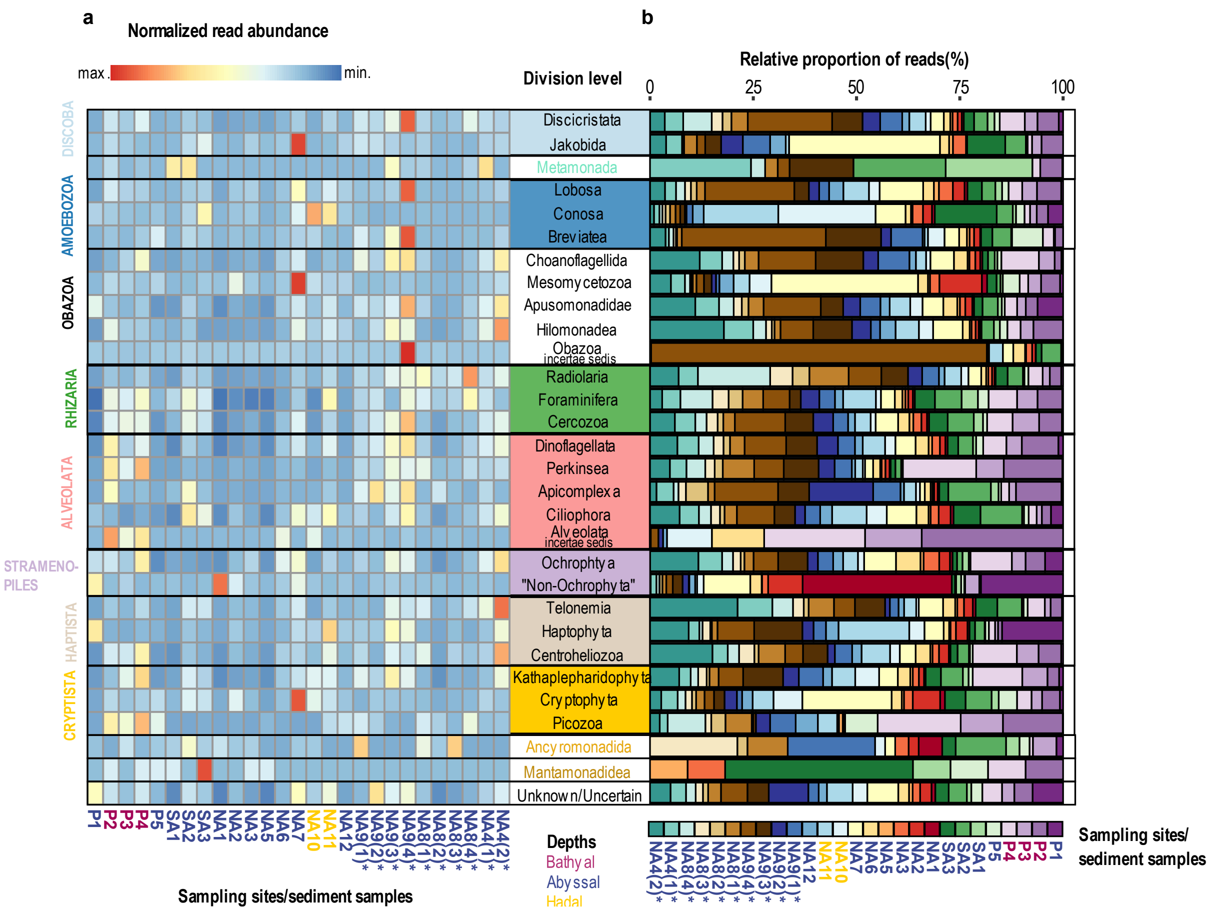

Supplementary Figure 5 | Read abundance of deep-sea heterotrophic protists. (a) Heatmap showing the read abundance of division levels per sampling site. Reads are normalized per division level by using the following function x-mean(x)/sd(x), where x is the sum of reads per division level considering all sampling sites. (**b**) Relative abundance (read number) within the 27 deep-sea sediment samples assigned to the major taxonomic protist groups on division level. Reads are set to 100% per division level considering all 27 sampling sites.

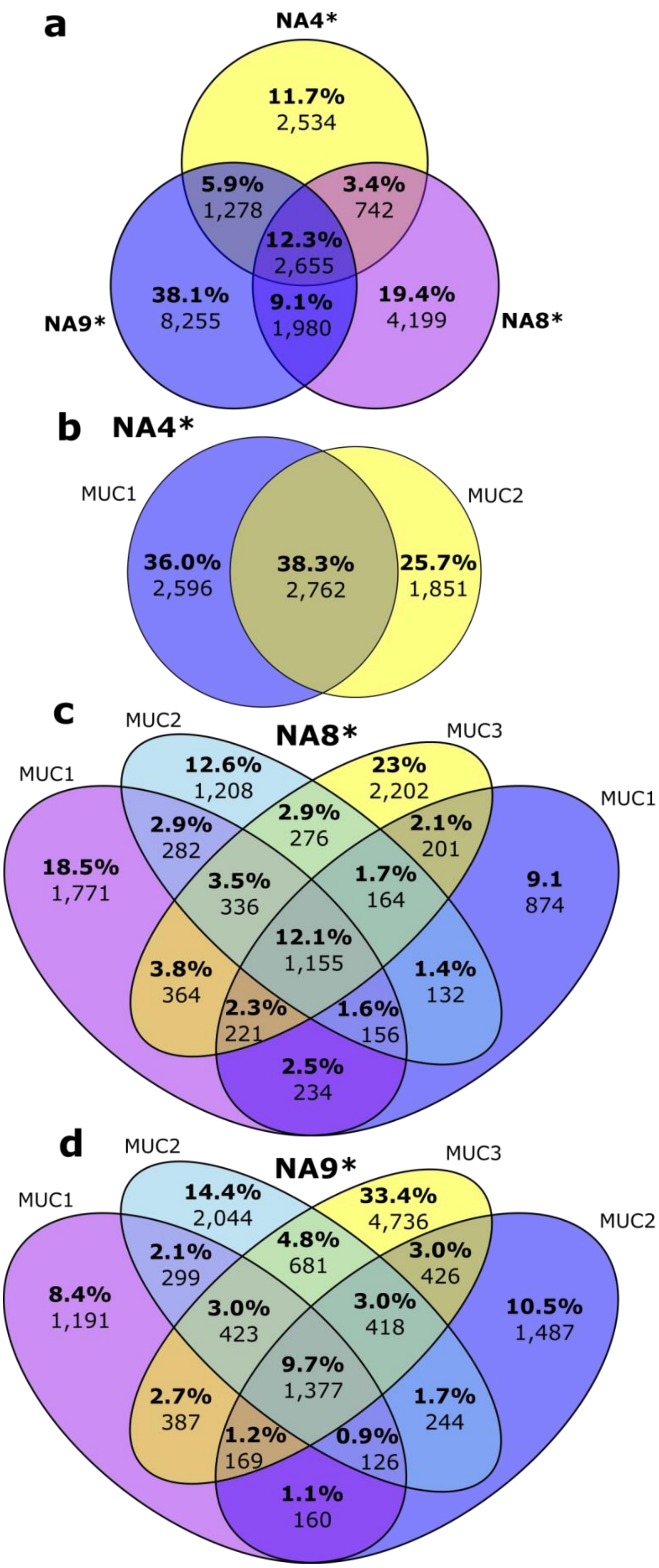

Supplementary Figure 6 | Small scale pattern of deep-sea heterotrophic protists. Number of shared and unique OTUs between (**a**) all three stations (inter-similarity, total richness = 21,643)) and per station (intra-station similarity) for (**b**) station NA9* (total richness = 14,168), (**c**) NA8* (total richness = 9,576) and (**d**) NA4* (total richness = 7,209) sampled during the deep-sea expedition (M139).

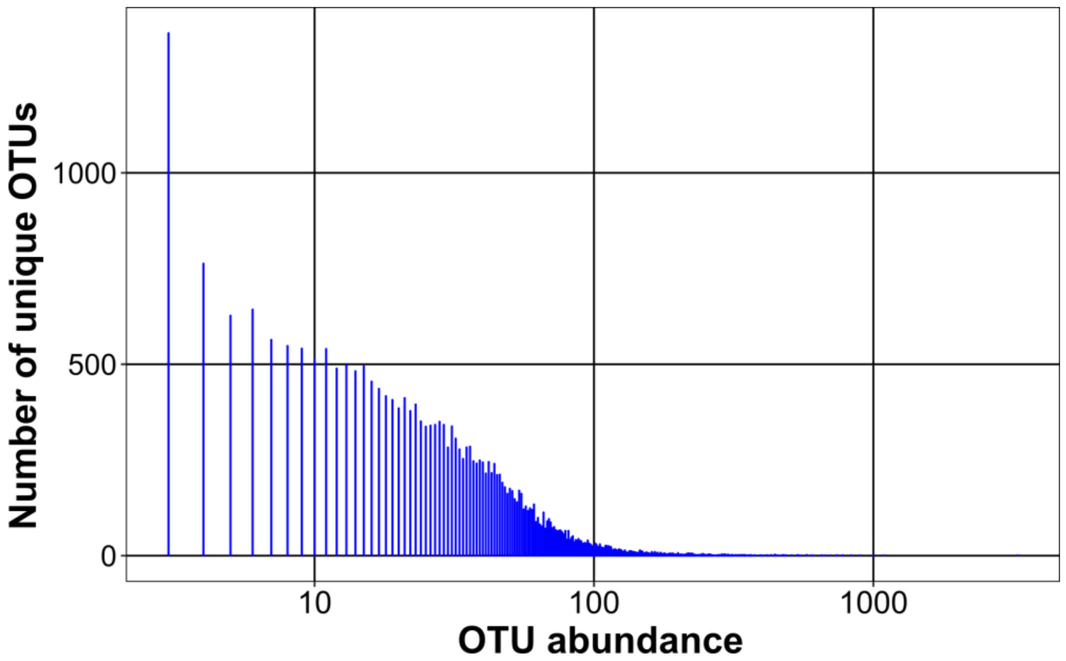

Supplementary Figure 7 | Unique deep-sea protist communities. Abundance of unique deep-sea heterotrophic protist OTUs. The x-axis is scaled logarithmically.

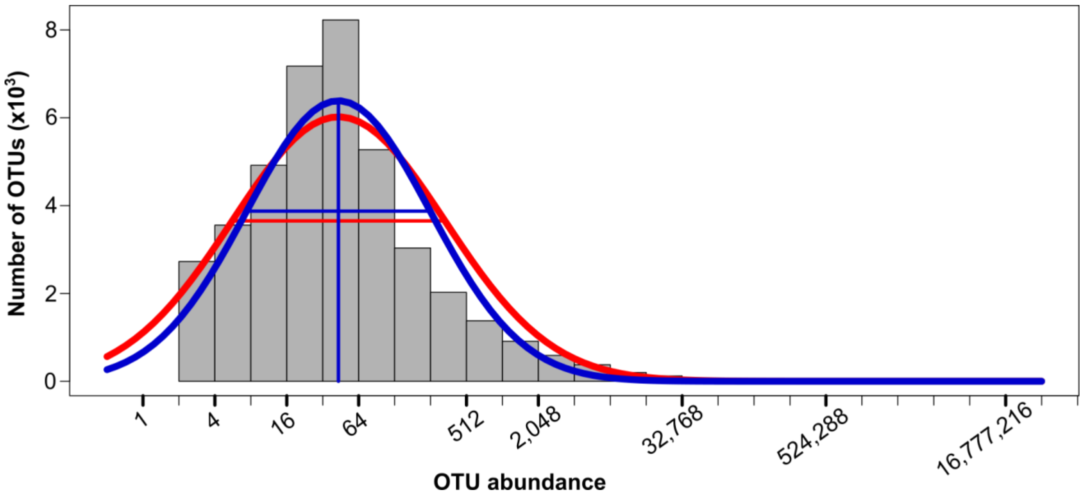

Supplementary Figure 8 | Deep-sea heterotrophic protist OTU abundance distribution. Most OTUs in our data set were represented by 16 to 64 reads. The OTU abundance distribution was fitted to the Preston-log normal model by using the Quasi-Poisson fit to octaves (red curve) and maximized likelihood to $\log_2$ abundances (blue curve) approximations.

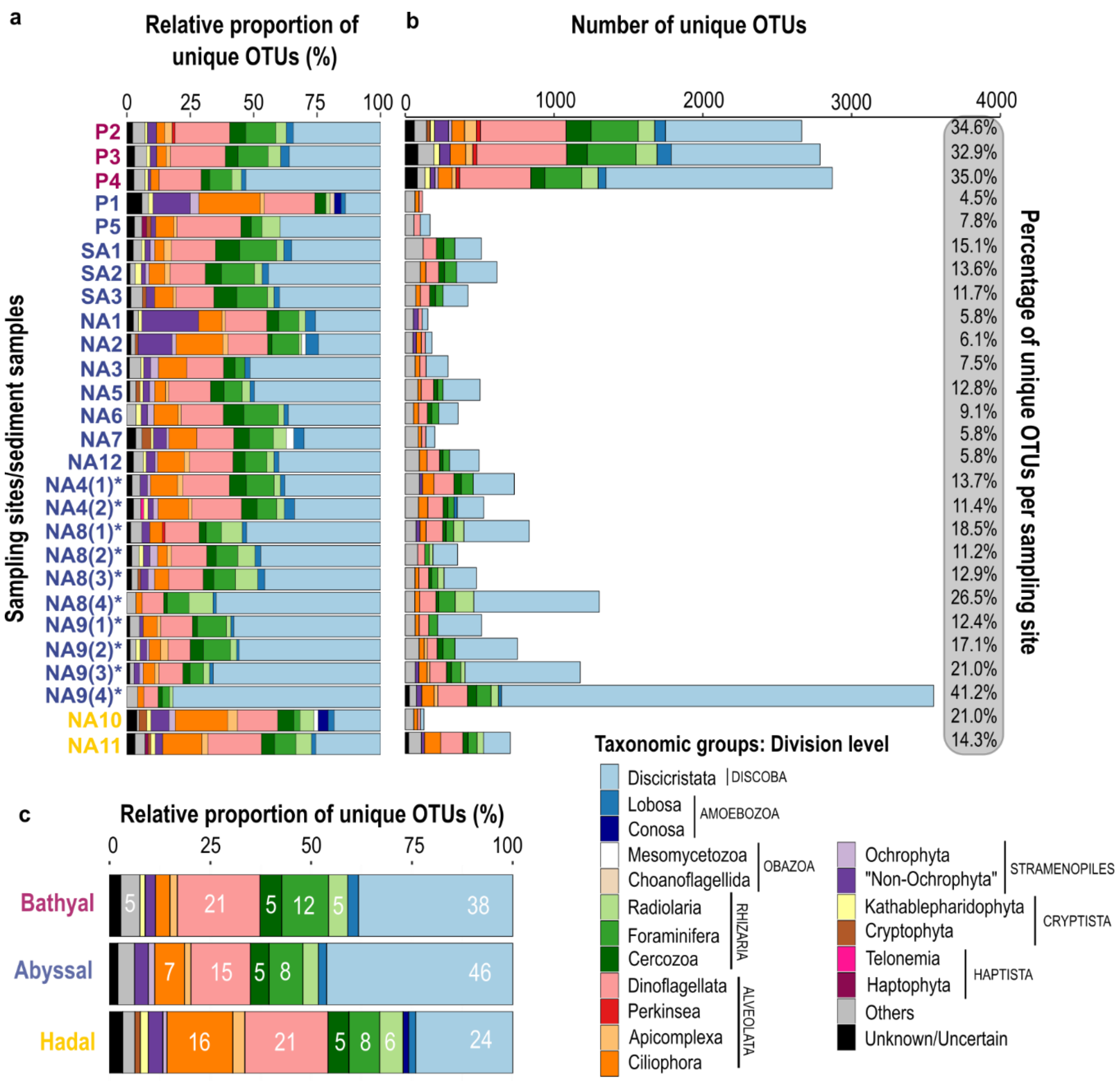

Supplementary Figure 9 | Unique deep-sea heterotrophic protist richness amongst samples. (a) Relative proportion of unique OTUs for each of the 27 sediment samples obtained from 20 deep-sea sites (see Fig. 1). (**b**) Total abundance of unique OTUs for each of the 27 sediment samples. Numbers (%) beside bar charts represent the percentage of unique OTUs of the total number of OTUs in each sediment sample. (**c**) Relative proportion of unique OTUs within the three depth zones.

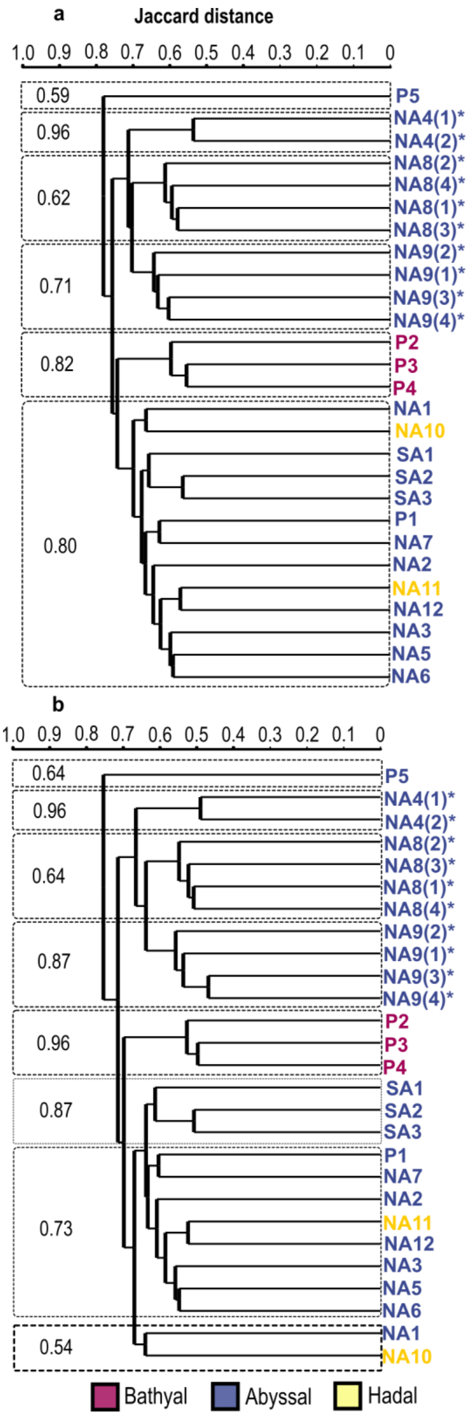

a

Sample	Number of OTUs ≥ 50 reads	Number of OTUs ≥ 3 reads	Loss of OTUs [%]
P5	1732	2150	19%
NA4(1)*	3638	5358	32%
NA4(2)*	3328	4613	28%
NA8(2)*	2594	3137	17%
NA8(4)*	3484	4919	29%
NA8(1)*	3157	4519	30%
NA8(3)*	2856	3709	23%
NA9(2)*	3364	4407	24%
NA9(1)*	3340	4132	19%
NA9(3)*	4043	5612	28%
NA9(4)*	5178	8617	40%
P2	4782	7697	38%
P3	4867	8486	43%
P4	4545	8194	45%
NA1	2633	2046	22%
NA10	2060	2572	20%
SA1	2559	3388	24%
SA2	3218	4523	29%
SA3	2727	3621	25%
P1	2056	2571	20%
NA7	2756	3436	20%
NA2	2446	2960	17%
NA11	3701	4947	25%
NA12	3574	4650	23%
NA3	3020	3848	22%
NA5	3013	3916	23%
NA6	3132	3896	20%

b

Sample	Number of OTUs ≥ 100 reads	Number of OTUs ≥ 3 reads	Loss of OTUs [%]
P5	1600	2150	26%
NA4(1)*	3130	5358	42%
NA4(2)*	3024	4613	34%
NA8(2)*	2270	3137	28%
NA8(3)*	2516	2856	32%
NA8(1)*	2743	4519	39%
NA8(4)*	2858	3709	23%
NA9(2)*	2872	4407	39%
NA9(1)*	2834	4132	31%
NA9(3)*	3404	5612	39%
NA9(4)*	3698	8617	57%
P2	3851	7697	50%
P3	4143	8486	51%
P4	3811	8194	53%
SA1	2313	2046	32%
SA2	2766	4523	39%
SA3	2432	3621	33%
P1	1905	2571	26%
NA7	2533	3436	26%
NA2	2233	2960	25%
NA11	3250	4947	34%
NA12	3185	4650	32%
NA3	2728	3848	29%
NA5	2803	3916	28%
NA6	2805	3896	28%
NA1	1893	2633	28%
NA10	1895	2572	26%

Supplementary Figure 10 | Similarity of deep-sea protist communities. Incidence-based clustering (Jaccard index) of heterotrophic protist community richness at the 27 sediment samples obtained from 11 deep-sea basins/regions visualized in dendrograms to investigate the impact of low abundant OTUs on clustering. (**a**) Only OTUs with ≥ 50 reads were considered (16,885 OTUs from 40,623 OTUs). (**b**) Only OTUs with ≥ 100 reads were considered (10,345 OTUs from 40,623 OTUs). The column "Loss of OTUs" shows the percentage of OTUs, which did not pass the filter criterion of either ≥ 50 reads or ≥ 100 reads per OTU, considering the total OTUs (containing ≥ 3 reads) per station.

Supplementary Table 1 | Downstream analysis of the deep-sea dataset. After merging of the paired reads and first filtering steps of our dataset including the removal of sequences without primers and sequences containing N's, we had a total of 181,441 OTUs and 203,500,576 reads. Number shown are OTUs and percentages as well as the number of reads and percentages of our second filtering approach including several filtering steps.

Filtering steps	No. OTUs	OTUs [%] from total	No. reads	Reads [%] from total
Total	181,441	100	203,500,576	100
Only Eukaryota	130,790	72.08	148,334,764	72.89
Chimeras excluded	117,477	64.75	148,081,692	72.77
Length - larger 87 bp	117,260	64.63	148,079,269	72.77
Quality > 0.0002	116,004	63.93	147,409,250	72.44
p-identity 80-100%	46,694	25.74	66,410,396	32.63
Fungi excluded	45,878	25.29	65,600,598	32.24
Metazoa excluded	43,346	23.89	57,773,422	28.39
Streptophyta excluded	42,929	23.66	57,341,089	28.18
Phototrophic protists excluded	40,623	22.39	55,283,811	27.17

Supplementary Table 2 | Deep-sea heterotrophic protist alpha-diversity. For each of the 27 separately analyzed sediment samples obtained from 20 deep-sea stations the total number of reads and OTUs after the final filtering is given. Different alpha-diversity indices were calculated per station.

Station Label	No. reads after filtering	No. OTUs after filtering	Shannon	Effective No of species	Simpson	Pielou	S.chao1
NA9*(1)	992,112	4132	6.55	699.33	1.00	0.79	5400
NA9*(2)	1,556,801	4407	4.94	140.46	0.91	0.59	5743
NA9*(3)	2,250,478	5612	5.16	173.93	0.90	0.60	6530
NA9*(4)	3,063,394	8617	5.85	345.73	0.97	0.65	9457
NA8*(1)	1,307,963	4519	5.04	155.15	0.93	0.60	5860
NA8*(2)	729,118	3137	4.76	116.34	0.91	0.59	4611
NA8*(3)	674,194	3709	5.87	353.82	0.98	0.71	4795
NA8*(4)	1,689,816	4919	4.83	124.99	0.93	0.57	5966
NA4*(1)	1,484,986	5358	5.39	218.54	0.92	0.63	6056
NA4*(2)	1,330,043	4613	6.47	648.06	0.99	0.77	5262
NA1	11,977,079	2633	0.13	1.14	0.02	0.02	4256
NA2	3,060,458	2960	1.05	2.85	0.21	0.13	4472
NA3	826,023	3848	3.56	35.13	0.67	0.43	4618
NA5	185,447	3916	6.15	466.71	0.98	0.74	4305
NA6	1,422,770	3896	3.24	25.45	0.62	0.39	5078
NA7	4,768,941	3436	2.08	7.98	0.42	0.26	4079
NA10	1,031,116	2572	3.50	33.13	0.76	0.45	4398
NA11	1,303,169	4947	6.16	471.46	0.99	0.72	5835
NA12	877,270	4650	4.05	57.39	0.73	0.48	5714
SA1	985,992	3621	5.25	189.72	0.93	0.64	4244
SA2	1,113,019	4523	5.92	372.93	0.98	0.70	5327
SA3	687,467	3388	3.45	31.56	0.68	0.42	4091
P1	6,845,141	2571	0.45	1.58	0.10	0.06	3927
P2	1,403,123	7697	6.80	896.34	0.99	0.76	8482
P3	1,868,307	8486	3.52	33.79	0.65	0.39	9155
P4	1,271,499	8194	6.55	698.58	0.99	0.73	9100
P5	578,085	2150	4.13	62.22	0.84	0.54	3806
