# Supplementary material for: High and specific diversity of protists in the deep-sea basins dominated by diplonemids, kinetoplastids, ciliates and foraminiferans
Source: Commun Biol. 2021 Apr 23;4:501. doi: 10.1038/s42003-021-02012-5 (PMC8065057; doi:10.1038/s42003-021-02012-5)
Supplement: Supplementary file 3 — Description of Additional Supplementary Files [file 42003_2021_2012_MOESM3_ESM.pdf]

## Description of Additional Supplementary Files

**File name:** Supplementary Data 1

**Description:** Detailed station list of sediment samples including information on each station (original names, expedition, depths, depth zone, latitude, longitude, year and region), fixation method and preparation of sediment samples for NGS analysis (sediment amount used for DNA extraction from different MUCs and Cores, pooling of DNA prior to PCR reaction, number of replicates in PCR) to investigate large- and small-scale distribution patterns.

**File name:** Supplementary Data 2

**Description:** List of sequenced (SSU rDNA V9-region) protist strains from the Heterotrophic Culture Collection (HFCC), ordered by classification of eukaryotes from[74]. Given are taxonomy, HFCC numbers, expedition name, region, sampling station, coordinates (latitude/longitude), sampling depth, sampling gear and the accession numbers for the whole 18S rDNA including the V9-region as well as references except for strains published within this study. MS: Mediterranean Sea; PO: Pacific Ocean; NAO: North Atlantic Ocean; FZ: Fracture Zone; BS: Baltic Sea; SAO: South Atlantic Ocean; IO: Indian Ocean; MUC: multi-corer; OW: overlaying water; Sed: sediment; Lat/Long: latitude/longitude; N.A.: not available.
