# Supplementary material for: High and specific diversity of protists in the deep-sea basins dominated by diplonemids, kinetoplastids, ciliates and foraminiferans
Source: Commun Biol. 2021 Apr 23;4:501. doi: 10.1038/s42003-021-02012-5 (PMC8065057; doi:10.1038/s42003-021-02012-5)
Supplement: Supplementary file 6 — Reporting Summary [file 42003_2021_2012_MOESM6_ESM.pdf]

nature research

Corresponding author(s): Alexandra Schoenle and Hartmut Arndt

Last updated by author(s): Feb 23, 2021

Reporting Summary

Nature Research wishes to improve the reproducibility of the work that we publish. This form provides structure for consistency and transparency in reporting. For further information on Nature Research policies, see our Editorial Policies and the Editorial Policy Checklist.

Statistics

For all statistical analyses, confirm that the following items are present in the figure legend, table legend, main text, or Methods section.

n/a	Confirmed	
☐	☒	The exact sample size (*n*) for each experimental group/condition, given as a discrete number and unit of measurement
☐	☒	A statement on whether measurements were taken from distinct samples or whether the same sample was measured repeatedly
☒	☐	The statistical test(s) used AND whether they are one- or two-sided<br>*Only common tests should be described solely by name; describe more complex techniques in the Methods section.*
☒	☐	A description of all covariates tested
☒	☐	A description of any assumptions or corrections, such as tests of normality and adjustment for multiple comparisons
☒	☐	A full description of the statistical parameters including central tendency (e.g. means) or other basic estimates (e.g. regression coefficient) AND variation (e.g. standard deviation) or associated estimates of uncertainty (e.g. confidence intervals)
☒	☐	For null hypothesis testing, the test statistic (e.g. *F*, *t*, *r*) with confidence intervals, effect sizes, degrees of freedom and *P* value noted<br>*Give P values as exact values whenever suitable.*
☒	☐	For Bayesian analysis, information on the choice of priors and Markov chain Monte Carlo settings
☒	☐	For hierarchical and complex designs, identification of the appropriate level for tests and full reporting of outcomes
☒	☐	Estimates of effect sizes (e.g. Cohen's *d*, Pearson's *r*), indicating how they were calculated

Our web collection on statistics for biologists contains articles on many of the points above.

Software and code

Policy information about availability of computer code

Data collection	Protist Ribosomal Reference database PR2 v4.11.1; Database W4 from the Tara Ocean project website (http://taraoceans.sb-roscoff.fr/EukDiv/#extraction)
Data analysis	downstream analysis of NGS raw data as described in https://github.com/frederic-mahe/swarm/wiki/Fred's-metabarcoding-pipeline and our material and methods part; Statistical analyses were conducted with R v.3.5.2 (packages: fossil, ggplot2, vegan, fpc)

For manuscripts utilizing custom algorithms or software that are central to the research but not yet described in published literature, software must be made available to editors and reviewers. We strongly encourage code deposition in a community repository (e.g. GitHub). See the Nature Research guidelines for submitting code & software for further information.

Data

Policy information about availability of data

All manuscripts must include a data availability statement. This statement should provide the following information, where applicable:

- Accession codes, unique identifiers, or web links for publicly available datasets
- A list of figures that have associated raw data
- A description of any restrictions on data availability

The data analyzed in this study are deposited at the Sequence Read Archive SRA (PRJNA635512), BioProject ID PRJNA635512, BioSamples SAMN15042370-SAMN15042370. The 18S rDNA sequences from 50 HFCC strains are deposited at GenBank under the Accession numbers MT355104-MT355153. Accession numbers of all 102 strains within our V9_DeepSea reference database can be found in the Supplementary Data 2. The deep-sea reference database V9_DeepSea can be downloaded from Zenodo (doi: 10.5281/zenodo.4305675).

Field-specific reporting

Please select the one below that is the best fit for your research. If you are not sure, read the appropriate sections before making your selection.

☐ Life sciences ☐ Behavioural & social sciences ☒ Ecological, evolutionary & environmental sciences

For a reference copy of the document with all sections, see nature.com/documents/nr-reporting-summary-flat.pdf

Ecological, evolutionary & environmental sciences study design

All studies must disclose on these points even when the disclosure is negative.

Study description	To explore protistan diversity in different deep-sea basins, we collected sediment samples from 20 sampling sites (3 bathyal sites, 15 abyssal sites, 2 hadal sites) in 11 regions in the Pacific and Atlantic Ocean. Besides sampling on a large scale to compare different deep-sea regions, we also investigated protist communities on a small spatial scale. See Supplementary Data 1 for more detailed information.
Research sample	During four different expeditions in the Pacific and Atlantic Ocean on board of the research vessels R/V Sonne (SO237, SO223T) and R/V Meteor (M79/1, M139) sediment samples from 20 different stations (3 bathyal, 15 abyssal, 2 hadal) at 11 deep-sea basins/ regions were collected using a Multi-Corer (MUC) (Supplementary Data 1). Sub-samples of the MUC-system were taken from the upper two mm sediment layer by means of a sterile syringe. Only tubes with undisturbed sediment and overlaying water were used for further analyses. For 17 stations (SA1-SA3, P1-P5, NA1-NA3, NA5-NA7, NA10-NA12) taken during expeditions SO237, SO223T and M79/1, three replicate sediment samples from three MUCs (corresponds to one core per MUC) were taken in total per station (Supplementary Data 1). For the three stations (NA4*, NA8*, NA9*) from the expedition M139, two to four replicates from three MUCs (corresponds to one to two cores per MUC) per station were taken (Supplementary Data 1). Samples were either fixated with 70% molecular biology graded ethanol and stored at -80°C or directly deep frozen at -80°C.
Sampling strategy	As many sediment samples as possible were inlcuded in the analysis, which have been sampled over the years. Depths of sampling and sequencing was determined by rarefaction curves for protist communities (see Supplementary Figure 2).
Data collection	Authors from this publication associated with the University of Cologne. Capt. Oliver Meyer, Uwe Pahl, Rainer Hammacher and the scientific and technical crews helped during sampling and supported us during the expeditions SO223T, SO237, M79/1 and M139.
Timing and spatial scale	Four different expeditions in the Pacific and Atlantic Ocean<br>R/V Sonne SO237 (Dec 2014/Jan2015), R/V Sonne SO223T (Sept/Oct 2012), R/V Meteor M79/1 (Jul/Aug 2009) , R/V Meteor M139 (Jul/Aug 2017)
Data exclusions	No data were excluded
Reproducibility	No experimental study. The upper 2mm sediment samples have been completly used for this study.
Randomization	Not applicable for this study.
Blinding	Not applicable for this study. Illumina sequencing was used to target the whole eukaryotic bidodiversity.

Did the study involve field work? ☒ Yes ☐ No

Field work, collection and transport

Field conditions	Temperature at the deep sea ranged between 2 and 4°C, salinity was about 36%oS. Detailed data on the conditions are available from published cruise reports: M139:https://doi.org/10.2312/cr_m139<br>M79.1; https://doi.org/10.2312/cr_m79_1 SO223T: urn: urn:nbn:de:gbv:46-00102872-14 SO237 doi 10.3289/ GEOMAR_REP_NS_23_2015.
Location	Due to the large sediment sample size please see Supplementary Data 1 for detailed information.
Access & import/export	Sediment samples were collected using a Multi-Corer (MUC). Most of the sediment was sampled at the open sea. Where samples were taken within EEZs, permission was obtained for each of the four deep sea expeditions (see Cruise reports of the four expeditions).
Disturbance	No disturbances during sampling.

Reporting for specific materials, systems and methods

We require information from authors about some types of materials, experimental systems and methods used in many studies. Here, indicate whether each material, system or method listed is relevant to your study. If you are not sure if a list item applies to your research, read the appropriate section before selecting a response.

Materials & experimental systems

n/a	Involved in the study
☒	☐ Antibodies
☒	☐ Eukaryotic cell lines
☒	☐ Palaeontology and archaeology
☒	☐ Animals and other organisms
☒	☐ Human research participants
☒	☐ Clinical data
☒	☐ Dual use research of concern

Methods

n/a	Involved in the study
☒	☐ ChIP-seq
☒	☐ Flow cytometry
☒	☐ MRI-based neuroimaging
